# Phylogenetic distribution and longitudinal persistence of plasmids in Mycobacterium abscessus

**DOI:** 10.1099/mgen.0.001807

**Published:** 2026-09-08

**Authors:** Trieu Le, Josephine M. Bryant

**Affiliations:** 1 Wellcome Sanger Institute, University of Cambridge, Cambridge, UK; 2 Centre for Evolution, University of Bath, Bath, UK

**Keywords:** *Mycobacterium abscessus*, NTM, pan-genome, plasmids

## Abstract

*Mycobacterium abscessus,* a non-tuberculous mycobacterium, is a cause of severe respiratory infections, notably in individuals with underlying lung conditions. Its high levels of intrinsic and acquired antimicrobial resistance make it particularly difficult to treat and horizontally acquired genetic elements may facilitate the spread of resistance. A small number of plasmids have been identified in this species, but their distribution, transmission dynamics across subspecies and clonal lineages remain poorly characterized. We analysed short-read genomic data from 3,060 *M*. *abscessus* isolates, including longitudinal samples, to characterize plasmid diversity and dynamics. Using a graph-based pan-genome approach, we identified 28 plasmids, including 15 previously unreported plasmids, mapped their distribution onto the species phylogeny and assessed their functional potential. Overall, 23.1% of isolates carried at least one plasmid, with higher prevalence in dominant circulating clones (DCCs) compared with non-DCCs. Plasmid carriage varied across subspecies and clonal backgrounds, and plasmids encoded numerous genes which may be linked to bacterial adaptation. Several plasmids persisted across multiple time points within individual patients, suggesting they can be highly stable over the course of a chronic infection.

**Impact Statement**

We discovered 15 novel plasmids in *Mycobacterium abscessus* using an enhanced detection approach that improves upon existing methods. These previously unrecognized plasmids substantially expand the mycobacterial plasmid database and uncover new layers of genetic diversity in this drug-resistant pathogen. Mapping these plasmids onto a global phylogeny revealed structured distribution patterns, suggesting a role in lineage-specific adaptation and transmission. Notably, we observed that plasmids persist across longitudinal patient samples, highlighting their long-term stability and likely importance in chronic infections. This study describes a novel bioinformatic approach to plasmid discovery as well as new biological insights into this important pathogen.

## Data Summary

The authors confirm all supporting data and protocols have been provided within the article or through supplementary data files.

## Introduction

*Mycobacterium abscessus* is an emerging opportunistic pathogen responsible for severe respiratory infections, particularly in individuals with cystic fibrosis (CF) and chronic obstructive pulmonary disease [1, 2]. Treatment is highly challenging due to its extensive intrinsic and acquired antibiotic resistance [3, 4]. Genomic analyses have classified *M. abscessus* into three subspecies: *M. abscessus* subspecies *abscessus* (*M. a. abscessus*), *M. abscessus* subspecies *massiliense* (*M. a. massiliense*) and *M. abscessus* subspecies *bolletii* (*M. a. bolletii*) [5]. Although once thought to be acquired exclusively from the environment, recent evidence indicates that *M. abscessus* can also be transmitted person-to-person, particularly among individuals with CF [5]. Whole-genome sequencing of isolates has identified globally dispersed dominant circulating clones (DCCs), suggesting adaptation to the human lung environment and transmission [6, 7].

Plasmids are key drivers of bacterial adaptation, facilitating horizontal gene transfer (HGT) and carrying traits that enhance survival in diverse environments [8–10]. In *M. abscessus*, for the small number of plasmids described, their distribution has been observed to be highly clonal, suggesting a fitness advantage to their hosts. For example, a putative plasmid, pMAB02, identified in a clonal strain of *M. a. massiliense* during a nationwide epidemic of surgical site infections in Brazil [11], encodes an *esx* gene cluster for a type VII secretion system potentially involved in conjugation [12] but has never been detected in any other isolates outside of this outbreak. Comparative genomic studies further indicate that, beyond chromosomal ESX loci, mycobacteria harbour diverse plasmid-encoded ESX systems that have undergone extensive diversification and may represent major drivers of mycobacterial adaptation and pathogenicity [13]. Plasmids have also been implicated in environmental adaptation, such as mercury resistance, although plasmid loss during chronic infection highlights potential fitness costs of plasmid maintenance [14]. Beyond *M. abscessus*, plasmids in other nontuberculous mycobacteria (NTM) have been linked to macrolide resistance in *Mycobacterium chelonae* [15] and associated with mercury and copper adaptation *in Mycobacterium marinum* and *Mycobacterium scrofulaceum* [16–18]. The close relatedness of plasmids identified across different NTM suggests that these elements may undergo interspecies transfer [19], thereby potentially contributing to the plasmid diversity observed within *M. abscessus*.

Despite these advances, the distribution, diversity and functional impact of plasmids in *M. abscessus* remain poorly characterized, leaving critical gaps in understanding their roles in adaptation, transmission and clinical outcome. The genetic background of different subspecies and clones may shape plasmid content, stability and function. In addition, variation in plasmid composition could have major implications for antibiotic resistance and pathogenicity. A comprehensive investigation of plasmid diversity and function is, therefore, essential to elucidate their contribution to *M. abscessus* evolution.

## Methods

### Data collection

We assembled a dataset of 3,060 *M*. *abscessus* genomes from multiple publicly available projects, including PRJEB2779 [7], PRJNA319839 [20], PRJNA398137 [21], PRJNA354248 [22], PRJNA439313 [23], PRJEB31559 [24], PRJEB37730 [25], PRJNA734660 [26], PRJDB10566 [27], PRJNA734909 [28], PRJNA832057 [29], PRJDB10333 [30], PRJDB13427 [31], PRJEB9515 [32], PRJNA315990 [33], PRJNA231221 [34], PRJEB7058 [11] and other studies with smaller datasets [35, 36].

The dataset represents 2,042 patients from 19 countries across five continents (Fig. S1, available in the online Supplementary Material). Most samples (95%) were derived from respiratory sources, such as sputum and bronchoalveolar lavage fluid, while the remainder originated from tissue, blood, wound and urine specimens. Longitudinal sampling was available for 304 patients. All genomes were short-read sequenced using Illumina platforms, which were taxonomically classified with Kraken v2.1.2 against a RefSeq-based database [37]; samples with fewer than 80% *M*. *abscessus* reads were excluded to reduce potential contamination (Fig. S2).

### Subspecies identification

For subspecies identification, sequencing data from each sample were compared to reference genomes of *M. a. abscessus* (CU458896.1), *M. a. massiliense* (GCF_001792625.1) and *M. a. bolletii* (GCF_003609715.1) using Mash v2.1.1 [38]. Genomic similarity was assessed through MinHash Jaccard estimation with default parameters (sketch size: 1,000; k-mer size: 21), and subspecies were assigned based on the highest shared-hashes score [39]. This method showed high concordance with previously published subspecies assignments for isolates originating from datasets analysed in that study.

### Phylogenetic tree construction and cluster identification

Sequencing reads were aligned to their respective reference genomes, as identified by Mash, using BWA-MEM v0.7.17 [40]. Phylogenetic trees were reconstructed with FastTree v2.1.10 [41] under default settings and then visualized using the ggtree R package v3.12.0 [42].

To infer genetic population structure, FastBAPS v1.0.8 [43] was applied to *M. a. abscessus* and *M. a. massiliense* using level selection 1 with hyperparameters of 0.012 and 0.011, respectively. Cluster assignments were further annotated with DCC labels from Bryant *et al.* [7], providing additional epidemiological context.

### Pan-genome construction from *de novo* assemblies

*De novo* genome assembly was performed using Shovill v1.1.0 with SPAdes as the underlying assembler, applying default settings [44]. Assembly quality was assessed using QUAST v5.0.2 [45], and genome annotation was carried out with Prokka v1.14.5 [46].

Pan-genome analysis was conducted using Panaroo v1.3.4 [47] in sensitive mode, separately for each genetic cluster and subspecies. Only samples from the earliest time point per patient were included. Singleton genes present in only one isolate were excluded from downstream analysis.

### Genomic diversity analyses

Pan-genome accumulation and diversity were evaluated using rarefaction curves based on Roary-format gene presence/absence tables from Panaroo. Analysis was performed with the rarefaction function in micropan v2.1 [48] using 100 random subsamplings without replacement. Median values were plotted for *M. a. abscessus* and *M. a. massiliense*.

### Plasmid identification

We initially used PlasmidFinder to identify putative plasmid-associated sequences [49], applying blastn [50] identity and coverage thresholds of 95 and 80%, respectively. However, its performance was limited with *M. abscessus* data.

To overcome this, we employed a graph-based approach to visualize the pan-genome using Cytoscape v3.10.0 [51]. To ensure clarity and reduce complexity, we utilized a reference genome for each subspecies as a guide in the visualization process. This method enabled us to effectively explore and analyse clusters within the accessory genomes. Clusters of interest were subjected to blast searches against the NCBI database to determine their identities and potential functions, and the complete contigs harbouring these clusters were extracted from the genome assemblies.

Candidate plasmids were evaluated using multiple lines of evidence, including the presence of plasmid-associated maintenance, partitioning, recombination and mobilization genes, such as *parA*/*soj* partitioning proteins, Hin recombinases, toxin–antitoxin systems and ESX secretion system components. Where applicable, sequence similarity to previously reported mycobacterial plasmids was assessed using blastn. Additional support was provided by the recovery of the same plasmids in multiple independent isolates.

To assess circularity, sequencing reads were mapped back to representative plasmid assemblies using BWA-MEM. Putative plasmids were linearized at an arbitrary position and paired-end reads spanning the junction between the start and end of the sequence were identified. Read pairs were considered supportive of circularity when one mate mapped within 200 bp of the start of the sequence and the other within 200 bp of the end. Coverage statistics and counts of junction-spanning read pairs were calculated for representative plasmids.

Plasmid functions were inferred from Prokka annotations and supplemented with the NCBI Prokaryotic Genome Annotation Pipeline for known plasmids. Plasmid gene synteny was assessed using Clinker [52], and visualization was performed using A Plasmid Editor v3.1.5 [53]. To evaluate plasmid persistence, blastn and mapping coverage analyses were conducted across longitudinal samples from 304 patients.

## Results

### Genomic diversity

**Fig. 1.**
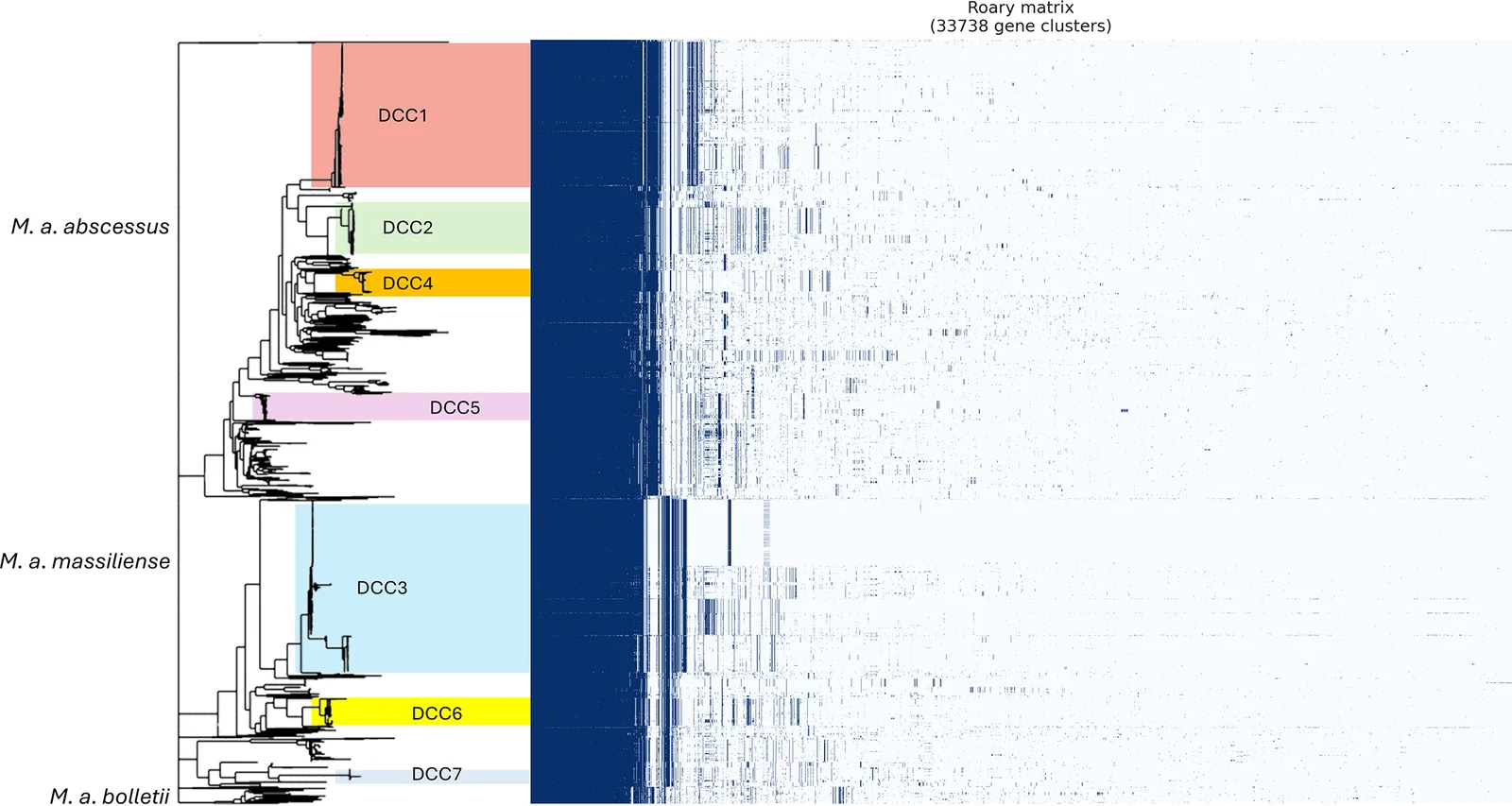
Gene presence/absence heatmap of *M. abscessus* isolates. A maximum-likelihood phylogeny of *M. abscessus* is represented on the left, with colours highlighting isolates within DCC1-7 and a heatmap showing gene presence (dark blue) or absence (light blue) is on the right. Each row shows the gene profile of each isolate.

Our pangenome analysis with panaroo [47] (Fig. 1) suggested that core genes accounted for ∼10.9% of the pan-genome, with 3,685 genes present in at least 95% of the isolates out of a total of 33,738 genes. This proportion of core genes is similar to that reported in previous studies, whereas the substantially larger pangenome likely reflects the broader sampling of our dataset, which enabled recovery of a wider accessory gene repertoire [35, 54–56]. We found the core genomes of *M. a. abscessus* and *M. a. massiliense* to be clearly differentiated, as shown by the long branch separating them in the maximum-likelihood phylogeny and network (Fig. 1).

The rarefaction curve displayed a steep initial rise followed by a plateau, indicating that the number of genomes analysed is sufficient to reliably assess the accumulation and diversity of the pan-genome of *M. abscessus* (Fig. S2).

### Plasmid diversity and distribution

Using PlasmidFinder, we detected the plasmid IncP-1β R751 (Accession: U67194), originally described in *Escherichia coli* and known for its broad host range and ability to replicate and transfer across diverse bacterial species [57, 58], in 124 DCC3 isolates associated with a surgical outbreak in Brazil [11, 59]. This plasmid could correspond to pMAB01, which was previously reported from the same outbreak [60]. Additionally, we detected plasmids in three other isolates whose sequences matched multiple plasmid replicons in the database (Table S1). Despite these findings, we failed to identify previously described *M. abscessus* plasmids using PlasmidFinder, indicating potential limitations in the detection tool and the lack of mycobacterial plasmids in the available databases. Consequently, our study shifted its focus to plasmid identification through a pan-genome analysis approach.

**Fig. 2.**
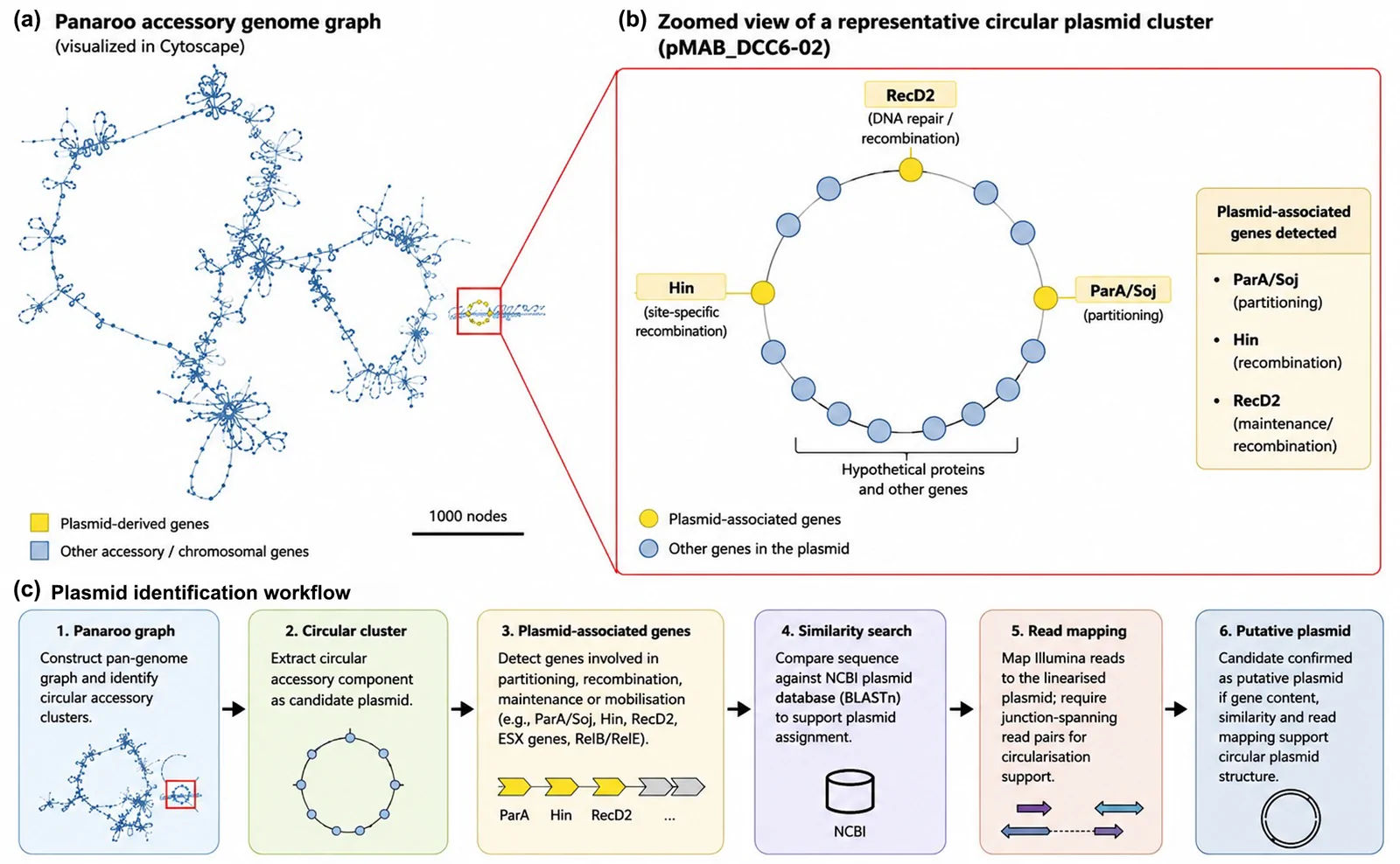
Workflow for plasmid identification using graph-based pan-genome analysis. (**a**) Accessory genome graph generated using Panaroo and visualized in Cytoscape. Nodes represent gene clusters and edges represent adjacency relationships in the pan-genome graph. Circular accessory genome components were identified as candidate plasmids. The highlighted region corresponds to a representative plasmid cluster. (**b**) Zoomed view of a representative plasmid cluster (*pMAB_DCC6-02*). The circular structure contains plasmid-associated genes involved in partitioning (*parA/soj*), recombination (*hin*) and maintenance (*recD2*), together with additional hypothetical proteins. These features are commonly associated with plasmid stability, persistence and mobility in bacteria. (**c**) Plasmid identification workflow. Candidate plasmids were identified as circular accessory genome components, evaluated for the presence of plasmid-associated genes, compared against known plasmids using blastn and further assessed through read-mapping analyses. Circularity was supported by paired-end reads spanning the junction between the start and end of the linearized plasmid sequence. Plasmids were classified based on multiple lines of evidence, including gene content, sequence similarity to known plasmids, recurrence across independent isolates and read-mapping support.

We identified 28 plasmids among the clinical *M. abscessus* isolates using pan-genome method (Fig. 2) and found that 23.1% of isolates carried at least one plasmid. Of these, 15 plasmids did not map to any previously reported plasmids. Eleven of the plasmids matched those previously reported in *M. abscessus*; these plasmids show higher prevalence compared to unreported plasmids. Additionally, two plasmids matched those previously reported in other species: one corresponding to FDAARGOS_1614, previously identified in *Mycobacterium kansasii* and another matching HPB1.1 plasmid unnamed2, found in *Mycolicibacterium aubagnense (formerly Mycobacterium aubagnense*).

The plasmids varied considerably in size, with a median of 22,402 bp (Fig. S3). The largest plasmid identified was 145,297 bp and the smallest plasmid was 9,547 bp. Gene synteny comparison revealed that many plasmids share partially similar sequences with others (Supplement figure 4), suggesting the presence of common genetic elements or shared evolutionary histories among these plasmids.

The distribution of plasmids among different subspecies and DCCs of *M. abscessus* reveals notable differences in plasmid prevalence (Fig. S5). Overall, *M. a. abscessus* exhibits a higher proportion of plasmid-bearing isolates (45.8%) compared to *M. a. massiliense* (10.8%). Meanwhile, *M. a. bolletii* shows a much lower proportion, with only 4% of isolates carrying plasmids.

The distribution of plasmids also varies significantly between DCCs and non-DCC isolates. For *M. a. abscessus*, isolates from DCCs have a higher proportion of plasmid carriers (38.9%) compared to non-DCC isolates (26.2%) (Table 1). Notably, DCC4 isolates do not carry any plasmids. Among the DCCs, DCC2 stands out with the highest proportion of plasmid-bearing isolates (80%) and the highest number of plasmids per isolate (Kruskal–Wallis test, H=516.65, df=9, *P*-value <2.2e-16) (Fig. S6). Meanwhile, for *M. a. massiliense*, non-DCC isolates demonstrate a higher prevalence of plasmid carriage (21.5%) compared to DCC isolates (4.7%).

**Table 1.** Summary of plasmid presence across different clones and subspecies

Subspecies	Cluster	N	Plasmid	Isolates with plasmid (%)	Total plasmid count	Average plasmid per isolate
***M. a. abscessus***	DCC1	340	13	89 (26.17)	133	0.39
	DCC2	120	9	96 (80.00)	168	1.40
	DCC4	43	0	0	0	0.00
	DCC5	31	2	20 (64.52)	21	0.68
	Non-DCC	589	14	167 (28.35)	186	0.32
***M. a. massiliense***	DCC3	504	5	13 (2.58)	14	0.03
	DCC6	80	4	10 (12.50)	13	0.16
	DCC7	36	3	6 (16.67)	8	0.22
	Non-DCC	222	9	48 (21.52)	56	0.25
***M. a. bolletii***	Non-DCC	49	2	2 (4.00)	2	0.04

### The phylogenetic distribution of the plasmids suggests a high degree of vertical transmission

**Fig. 3.**
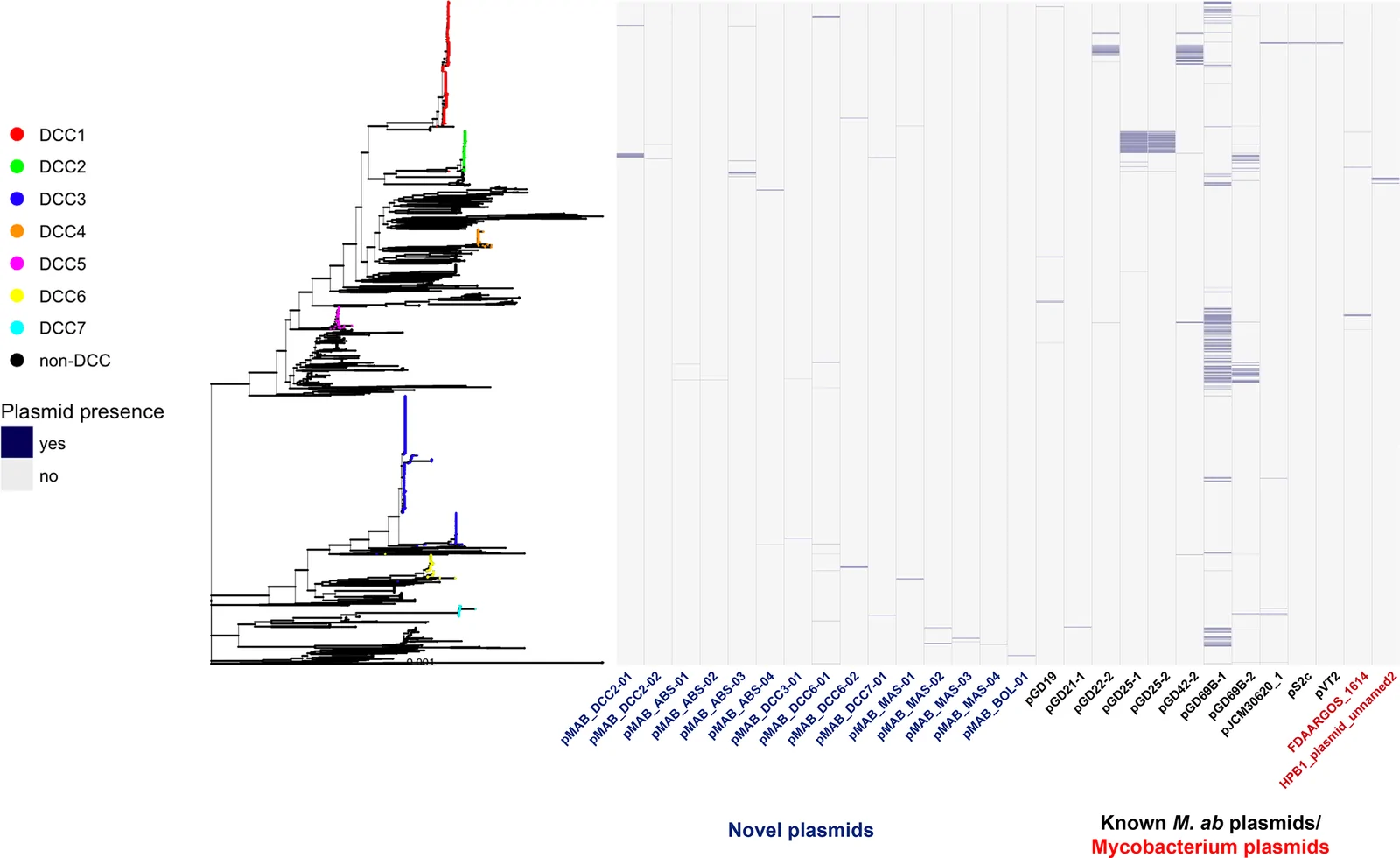
Heatmap of plasmid presence across the phylogenetic tree of *M. abscessus*. The phylogenetic tree, constructed using FastTree, is coloured to indicate DCC1-7 or non-DCC isolates. The heatmap shows plasmid presence in dark blue and plasmid absence in grey. The column on the left lists the plasmids identified in this study: blue text indicates previously unreported plasmids, black text denotes plasmids previously reported in *M. a. abscessus* and red text highlights plasmids found in other species.

**Fig. 4.**
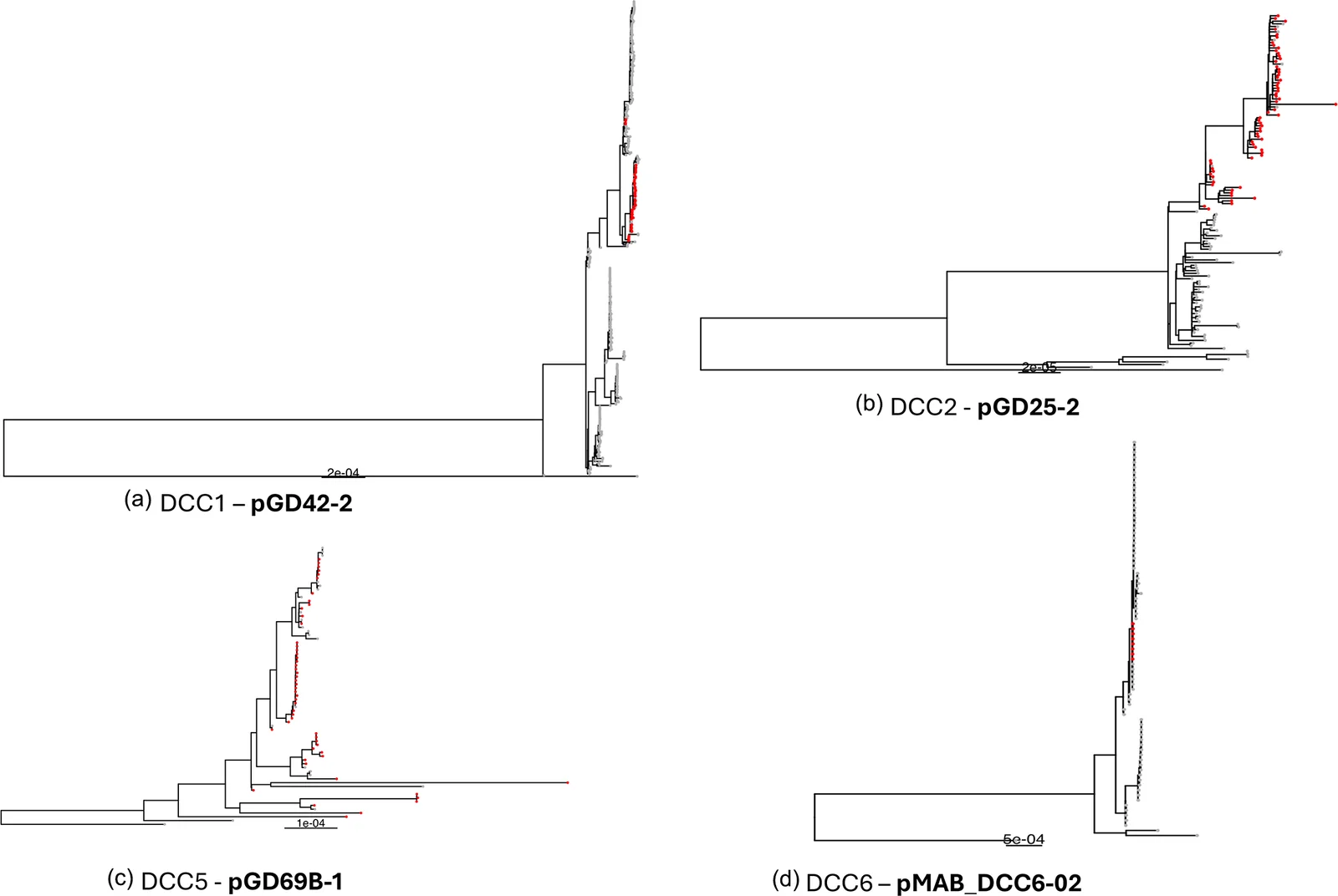
Representative examples of phylogenetic clustering of plasmid-positive isolates within DCCs. (**a**) Plasmid pGD42-2 in DCC1; (**b**) Plasmid pGD25-2 in DCC2; (**c**) Plasmid pGD68B-1 in DCC5; and (**d**) Plasmid pMAB_DCC6-02 in DCC6. Red tip points on the phylogenetic trees indicate the presence of the corresponding plasmids. These panels are shown as representative examples of the strong phylogenetic clustering observed for plasmid-positive isolates and do not imply that the corresponding plasmids are restricted to the illustrated DCCs.

We observed that isolates carrying the same plasmids often have close phylogenetic relationships (Fig. 3) with a non-random distribution restricted to a small number of clades, suggesting single acquisition events followed by vertical transmission. More detailed analysis of the DCCs further supports this scenario with a high degree of monophyly observed (Fig. 4). Among the plasmids found in this study, pGD69B-1 was the most common, found in 205 isolates (10.2%). In the DCC5 clade, 20 isolates (64.5%) carried pGD69B-1, though there was only a single instance of plasmid acquisition observed (Fig. 4c). Moreover, plasmid pGD69B-1 was identified across two subspecies and four DCCs, suggesting multiple acquisition or transmission events. Plasmid pGD42-2 was strongly associated with DCC1, with isolates carrying this plasmid clustering together on the phylogeny (Fig. 4a). A similar pattern was observed for pGD22-2. However, a small number of isolates outside DCC1 also harboured these plasmids, suggesting that they were transmitted through both vertical inheritance and horizontal transfer. Plasmids pGD25-1 and pGD25-2 were highly prevalent in the DCC2 clade, with 68 (56.7%) and 61 (50.1%) isolates carrying them, respectively. These plasmids also likely underwent vertical transfer among isolates within this clade (Fig. 4b). We also found that less common plasmids including pJCM30620_1, pS2c and pVT2 were restricted to a small number of closely related isolates within DCC1. In contrast, FDAARGOS_1614 was detected in a few isolates belonging to DCC5 and DCC2, suggesting that horizontal transfer of this plasmid may have occurred on more than one occasion.

### Potential functions of plasmids

**Fig. 5.**
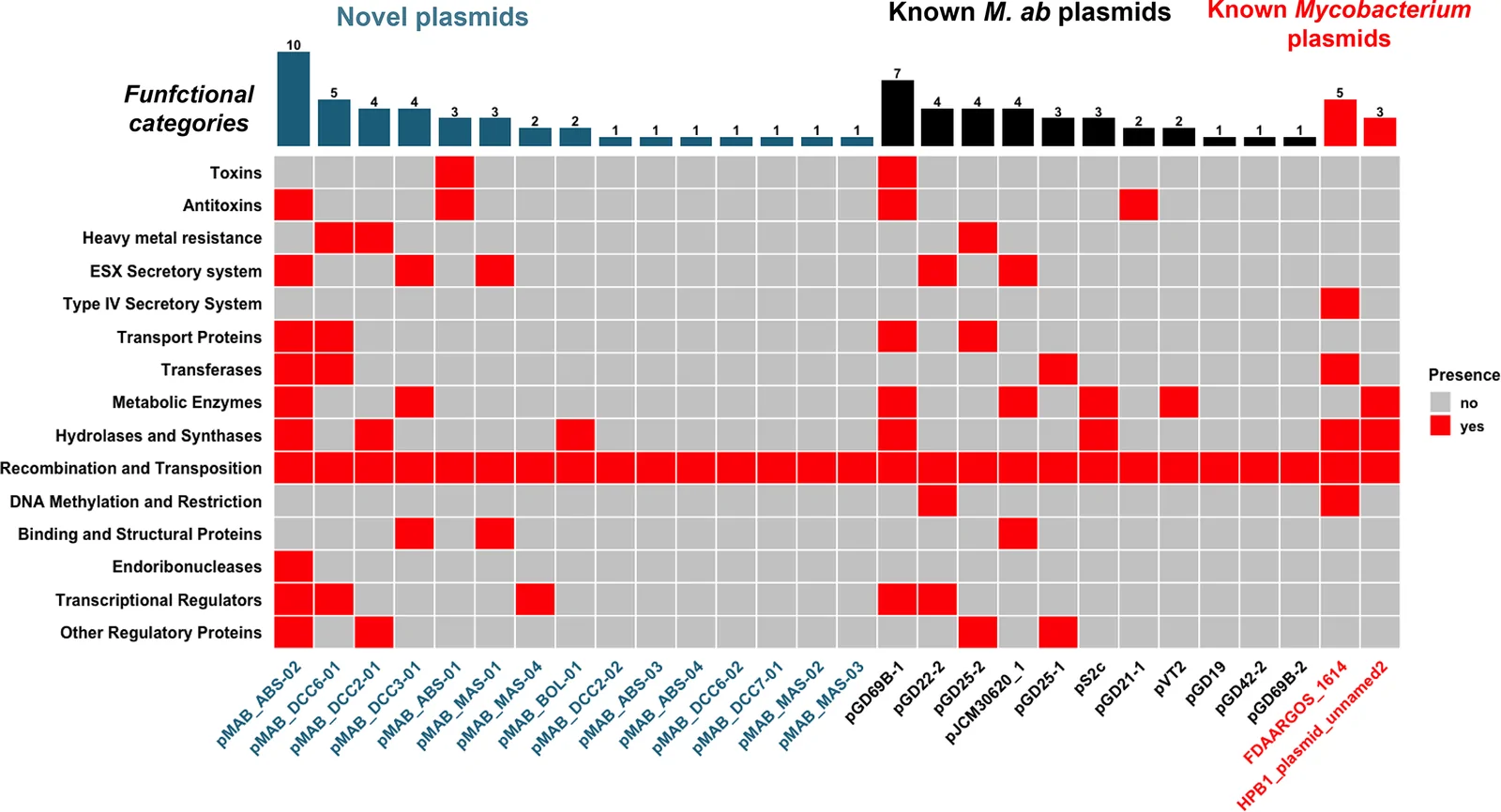
Heatmap of gene functions in identified plasmids. Gene functions were annotated using Prokka and supplemented with annotations from NCBI for previously reported plasmids. The y-axis represents the gene functions, while the x-axis lists the identified plasmids. Red indicates the presence of genes with the specified functions in the plasmids, whereas grey denotes the absence of these genes.

The plasmids detected in this study exhibit a diverse range of putative gene functions, (Fig. 5 and Table S2). As expected, genes associated with recombination and transposition, such as DNA invertase (*hin*), recD DNA helicase (*recD2*) and chromosome partitioning protein (*parA*), are present across all the plasmids.

The most abundant plasmid, pGD69B-1 features a range of functional genes (Fig. S7A), including several transcriptional regulators and metabolic enzyme genes. Additionally, it contains an Abi family protein associated with abortive infection systems, potentially providing defence against bacteriophage infection [61].

We identified several resistance and virulence genes in plasmids, including mercuric reductase (*merA*) for heavy metal resistance, cadmium resistance regulator (*cadC*) in pGD25-2 and thiol-disulfide oxidoreductase (*resA*), which aids survival under oxidative stress (Fig. S7B).

Several toxin and antitoxin systems were identified, including *relE*, *relB* and *relJ*, suggesting roles in plasmid stability and stress response. Antitoxins, such as *mazE* and *marF* were also found on these plasmids, which may further support plasmid maintenance by regulating toxin activity.

Multiple plasmids are predicted to carry ESX system genes linked to virulence and immune evasion. These ESX system genes have previously been identified as integral to mycobacteria’s ability to secrete virulence factors and evade host immune defences [62].

Additionally, mycosins-1, are notable proteins associated with ESX systems. They can serve essential roles in the secretion and function of virulence factors, contributing to the bacteria’s pathogenic capabilities [62].

The *M. aubagnense* plasmid HPB1_plasmid_unnamed2 carries genes linked to metabolism and redox regulation, including alx, which may help manage oxidative stress (Fig. S7C). The *M. kansasii* plasmid FDAARGOS_1614 carries genes with predicted functions for restriction, metabolism, gene regulation and conjugation, potentially aiding in adaptation, immune evasion and gene transfer (Fig. S7D).

### Longitudinal stability of plasmids

**Fig. 6.**
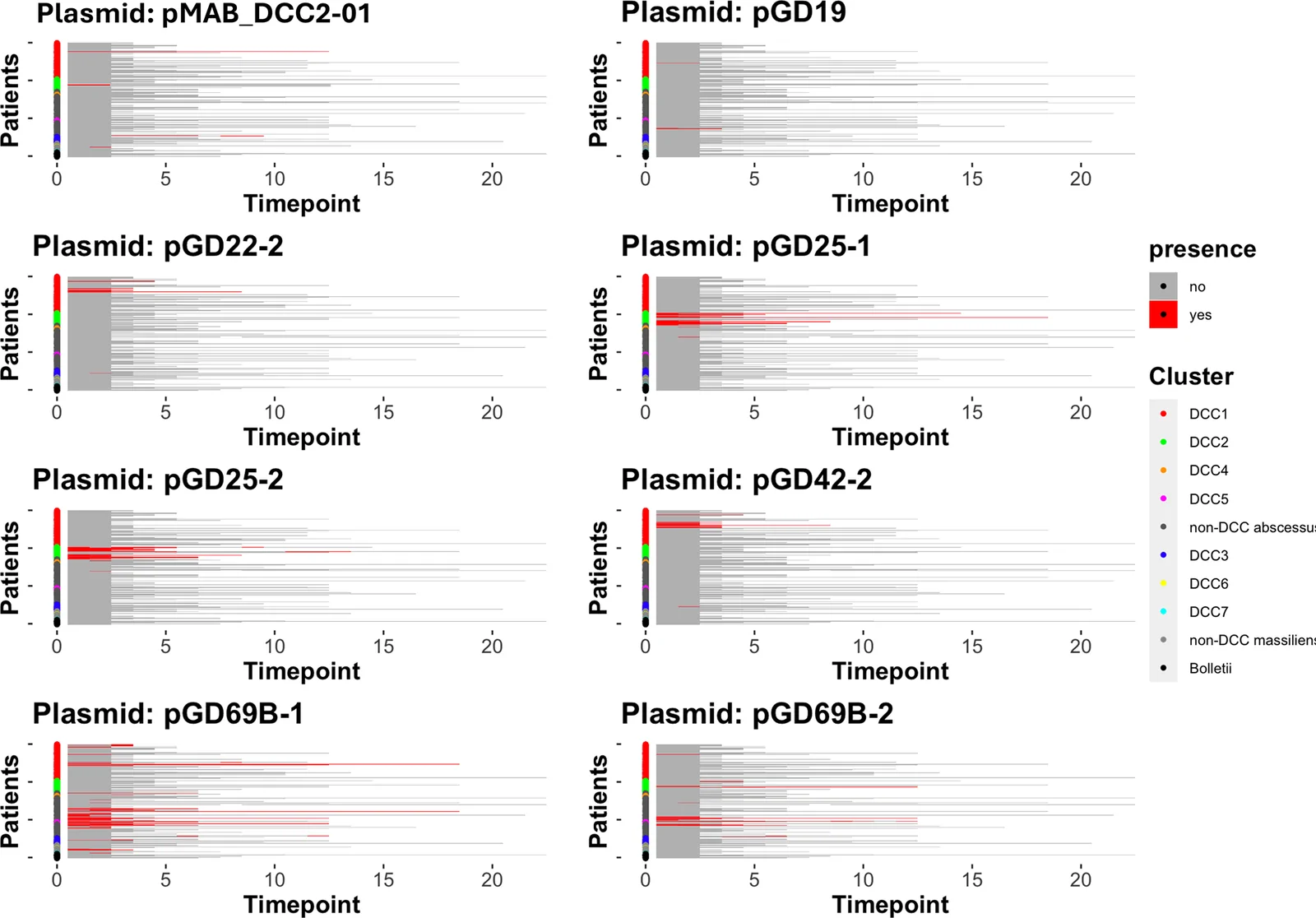
Heatmaps of plasmid presence across longitudinal samples. Each plot depicts the presence of the plasmid labelled correspondingly across longitudinal samples. Each row represents a patient with samples collected at different time points. The y-axis colours denote the DCC1-7 or non-DCC classification of the initial samples. The x-axis indicates the different time points. Red indicates the presence of plasmids, while grey indicates their absence. Sampling intervals varied between patients and were not uniformly available in the source datasets; therefore, the x-axis reflects the sequence of isolate collection rather than a standardized timescale.

Due to the chronic nature of respiratory *M. abscessus* infections, we were able to study plasmid stability over the course of long-term infection. Longitudinal sampling was available for 304 patients; however, only 147 patients had at least one isolate carrying a plasmid and were, therefore, included in the plasmid persistence analysis. Plasmid presence remained stable across longitudinal samples in 122 of these 147 patients (83%), as shown in Fig. 6 and Table S3. Many plasmids persisted in patients for more than 1 year. For example, pGD69B-2 persisted in three patients for more than a year, and pGD69B-1 persisted in five patients for 1–2 years. Notably, plasmid pGD25-1 showed high persistence in two patients with DCC2 isolates, remaining detectable for more than 2 years. In several cases, plasmids were absent at the initial time point but were detected after 1–2 months of treatment, including pGD69B-1, pGD69B-2, pGD25-1, pGD25-2, pGD22-2 and pMAB_DCC2_01. In one patient, plasmid pGD25-2 was present during the first 6 months, subsequently lost, and then detected again after 1 year of treatment. This suggests that subpopulations of the infecting pool have lost the plasmid whilst others have retained it.

## Discussion

In this study, we have catalogued the diversity of plasmids in *M. abscessus*, leveraging a globally sourced dataset spanning multiple continents to uncover patterns of plasmid distribution and mobility across the species’ worldwide phylogeny. The results revealed the presence of 28 plasmids of varying sizes and gene functions. Notably, many plasmids identified using our method have not been reported in previous studies [19, 63], although experimental confirmation of these putatively novel plasmids was beyond the scope of the present study. This highlights the limitations of standard tools like PlasmidFinder, which failed to detect them, underscoring the underrepresentation of mycobacterial plasmids in current databases. Updating these databases and adopting pan-genome graph-based approaches may improve the detection of novel plasmids in mycobacterial species. The comprehensive approach in this study provides new insights into the evolutionary dynamics and geographical spread of plasmids in *M. abscessus*.

Plasmid distribution varies across subspecies and clones, with plasmids clustering on the phylogeny, indicating both horizontal and vertical gene transfer, consistent with observations in other bacteria [8, 64]. Shared regions among plasmids suggest that they have a common ancestry or potential plasmid fusion, but further investigation is needed. No clear association was observed between plasmid distribution and geographic origin, suggesting that plasmid presence is more strongly linked to the bacterial genetic background than to regional factors. Genetic studies have revealed a high degree of genetic variability within the *M. abscessus* complex, with considerable diversity among isolates collected from the same clinical sites and even among strains of the same subspecies [5, 65–67]. This genetic diversity could be facilitated by the presence of mobile genetic elements, such as plasmids, which mediate HGT and contribute to the spread of antibiotic resistance and other adaptive traits [68].

We found that *M. a. abscessus* has a higher proportion of plasmid-bearing isolates and a larger pan-genome than *M. a. massiliense*, suggesting more frequent HGT. Within *M. a. abscessus*, DCCs exhibit greater plasmid prevalence, except for DCC4. In contrast, non-DCC isolates in *M. a. massiliense* display higher plasmid diversity and proportions, suggesting that they may acquire new genetic materials via a different mechanism or inhabit a different ecosystem. Notably, two plasmids HPB1_plasmid_unnamed2 and FDAARGOS_1614 were originally detected in *M. aubagnense* and *M. kansasii*, showing that plasmid transfer can occur across different mycobacterial species [69, 70]. These findings underscore diverse patterns of plasmid presence across the *M. abscessus* complex.

Plasmids in *M. abscessus* are predicted to encode a broad range of functions which may play key roles in facilitating bacterial adaptation across diverse environments. High-prevalence plasmid pGD69B-1 is predicted to encode several transcriptional regulators, including those from the TetR/AcrR family, involved in multidrug resistance by controlling the expression of efflux pumps that expel toxic compounds, including antibiotics, out of the cell [71]. Additionally, pGD69B-1 encodes Abi family proteins associated with abortive infection systems, which provide defence against bacteriophage infections and contribute to the stabilization of mobile genetic elements, such as plasmids [61]. Heavy metal resistance genes are another important adaptive feature found in mycobacterial plasmids, particularly in plasmid pGD25-2 found in isolates of DCC2. pGD25-2 harbours genes like mercuric reductase (*merA*), which converts toxic mercuric ions into less harmful elemental mercury, and cadmium resistance transcriptional regulator (*cadC*), both of which confer resistance to heavy metals [16, 17]. These genes could enable mycobacteria to thrive in environments contaminated with heavy metals, which would otherwise be lethal.

Many of these plasmids contain genes that contribute to drug resistance via membrane activities. The ESX secretion systems, encoded on some mycobacterial plasmids, further enhance pathogenicity by providing additional or specialized secretion capabilities. These systems facilitate the secretion of virulence factors that aid in immune evasion, intracellular survival and host colonization [72]. The presence of ESX systems on plasmids also contributes to the genetic diversity and evolution of mycobacteria by allowing them to acquire new effector proteins and adapt to new hosts or environments. Mycosin-1 and Mycosin-5 are serine proteases associated with the ESX systems, playing vital roles in bacterial pathogenicity and adaptation. Mycosin-1 is essential for activating virulence factors like *EsxA*, which disrupts host immune defences [73], while Mycosin-5 is involved in the secretion of PE/PPE proteins that contribute to antigenic diversity [74]. In this study, these proteins were identified on only a small number of plasmids, highlighting the diverse functional repertoire carried by plasmids within the *M. abscessus* complex. Their occurrence illustrates the potential for rare plasmids to mobilize specialized functions that may influence adaptation and pathogenicity in specific genetic backgrounds.

These plasmids demonstrated stable presence in longitudinal isolates, with phylogenetic analysis supporting their persistence after acquisition. This highlights their potential role in bacterial adaptation. Notably, several plasmids appeared in later time point isolates but were absent in earlier ones from the same patients. This could be explained by acquisition during infection or treatment, possibly from environmental sources. However, we propose a more likely explanation of within-host subclonal diversity, where some subclones have lost the plasmid, but others have not. High levels of within-host subclonal diversity and the maintenance of this diversity over time are well established for chronic *M. abscessus* infections [6, 7]

This study offers valuable insights into plasmid dynamics in *M. abscessus*, though several limitations must be acknowledged. Our sampling could be biased towards clinical isolates, due to the limited data from environmental isolates, potentially skewing our understanding of the organism’s natural plasmid content and diversity. Additionally, the use of short-read data may lead to incomplete and fragmented pan-genome results, suggesting that long-read data could enhance accuracy [75]. The 15 putatively novel plasmids identified in this study could not be experimentally validated or confirmed using long-read sequencing, as all isolate genomes originated from publicly available datasets and the corresponding biological material was unavailable for further investigation. An additional limitation is that phylogenetic reconstruction was performed without genome-wide recombination filtering. Recombination has been reported in *M. abscessus* and may influence branch lengths and phylogenetic relationships. Although analyses of representative subspecies datasets using recombination-filtered alignments produced broadly concordant plasmid distribution patterns, residual effects of recombination may have influenced the inferred phylogenetic structure. Moreover, gene functional annotation in NTMs remains incomplete, with many plasmid-associated genes lacking characterization. The choice of annotation pipeline significantly impacts pan-genome predictions, with biases introduced by how pseudogenes are handled [75]. Future studies should incorporate more environmental isolates from diverse niches to better capture plasmid diversity. Improved annotation tools and deeper insights into gene functions are also essential to fully understand how these plasmids contribute to bacterial evolution.

## Supplementary material

10.1099/mgen.0.001807 Supplementary Material 1.

10.1099/mgen.0.001807 Supplementary Material 2.

10.1099/mgen.0.001807 Supplementary Material 3.

## Abbreviations

CF: cystic fibrosis
DCC: dominant circulating clone
HGT: horizontal gene transfer
NTM: nontuberculous mycobacteria
