# Supplementary material for: Phylogenetic distribution and longitudinal persistence of plasmids in Mycobacterium abscessus
Source: Microb Genom. 2026 Sep 8;12(9):001807. doi: 10.1099/mgen.0.001807 (PMC13552800; doi:10.1099/mgen.0.001807)
Supplement: Supplementary file 1 [file mgen-12-01807-s001.pdf]

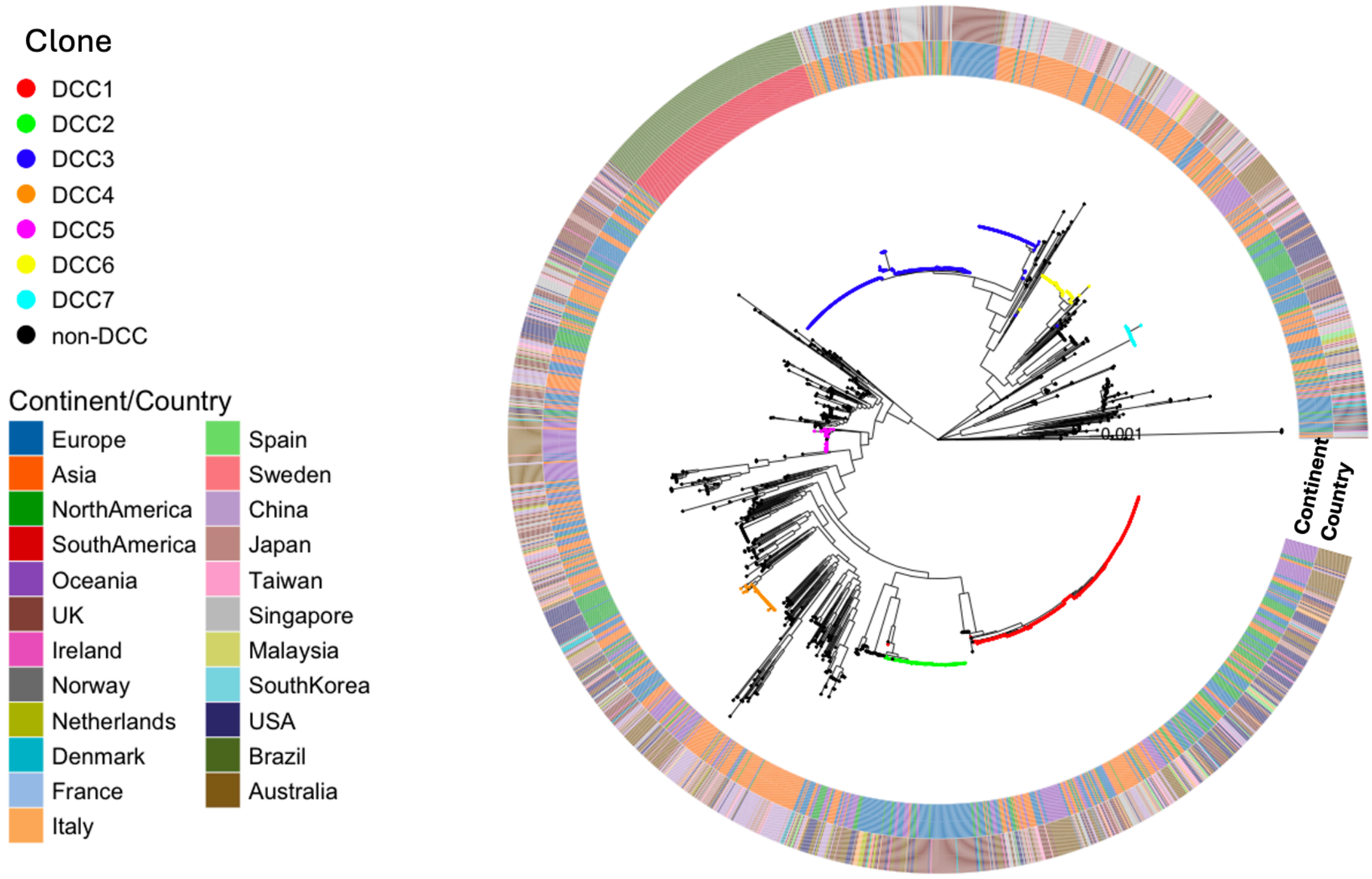

Supplementary figure 1. Geographical distribution of *M. abscessus* DCCs on phylogenetic tree. Phylogeny of 2,015 *M. abscessus* isolates with colours indicating DCC 1-7 or non-DCC. The inner and outer rings with colours indicate continents and countries, respectively, where the isolates were collected.

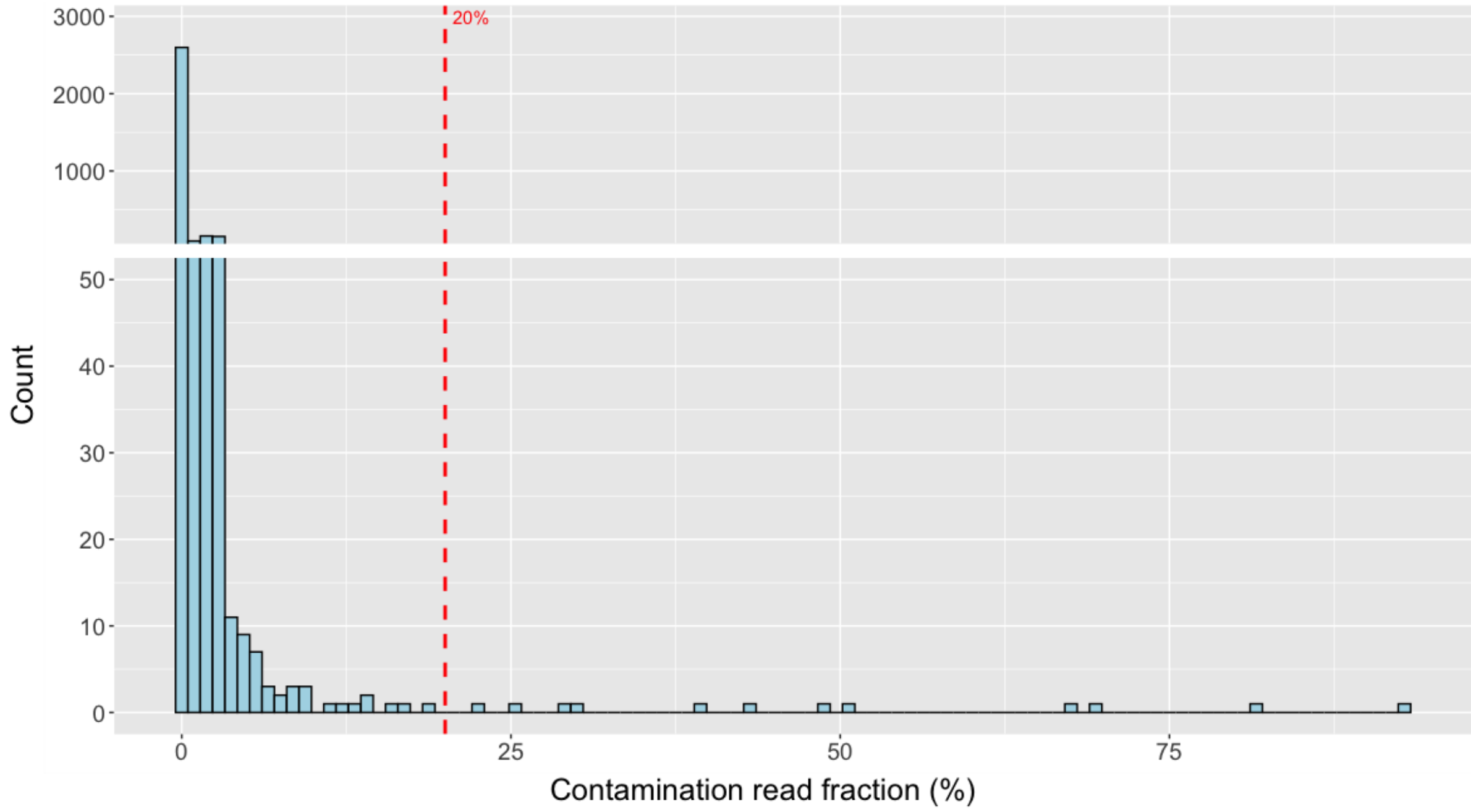

Supplementary figure 2. Distribution of contamination read fractions across isolates. The y-axis is broken between 50 and 200 isolates to facilitate visualization of both the highly abundant low-contamination isolates and the sparse high-contamination tail. The red dashed line denotes the 20% contamination threshold used for quality control, above which isolates were excluded from downstream analyses.

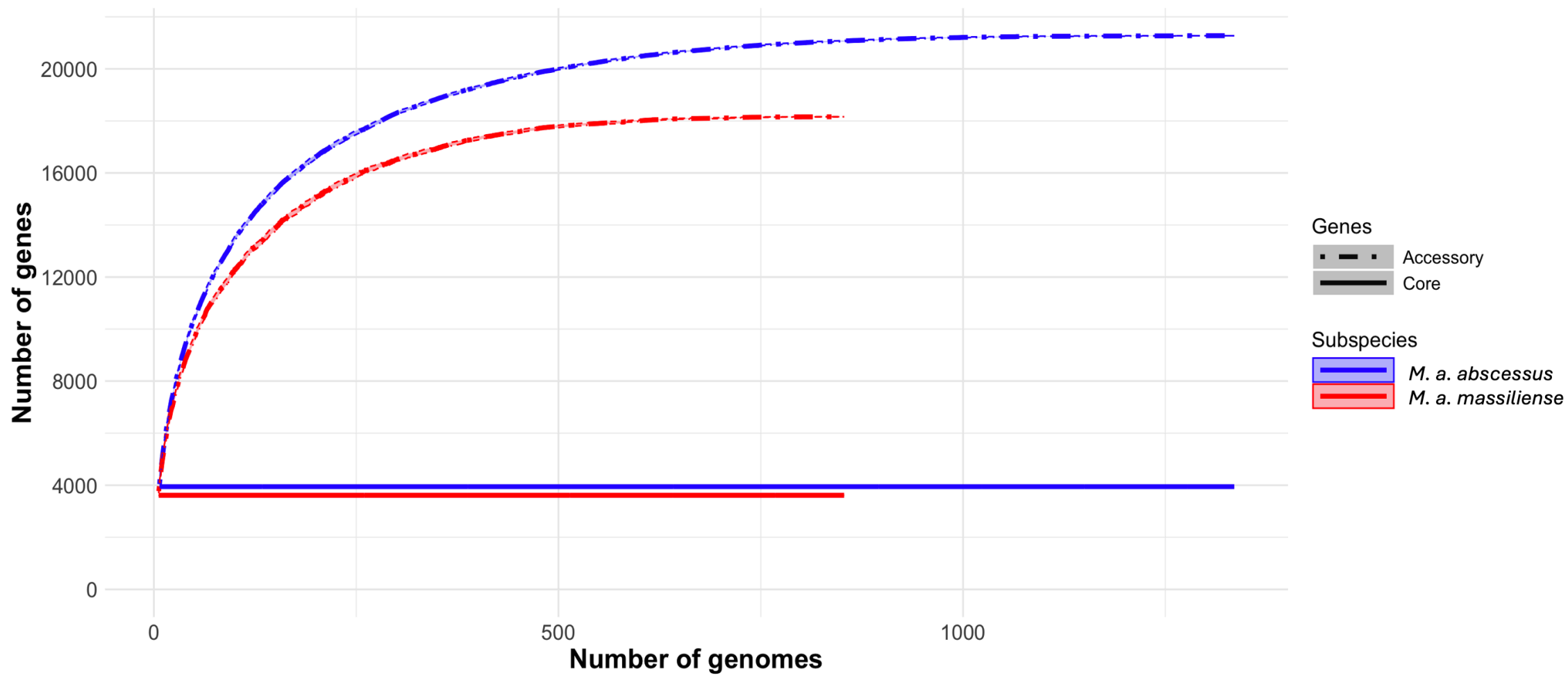

Supplementary figure 3. Rarefaction curves illustrating the relationship between the number of genes and the number of genomes. Solid lines represent core genes present in more than 95% of isolates, while dashed lines represent accessory genes. The blue line corresponds to *M. a. abscessus* and the red line to *M. a. massiliense*.

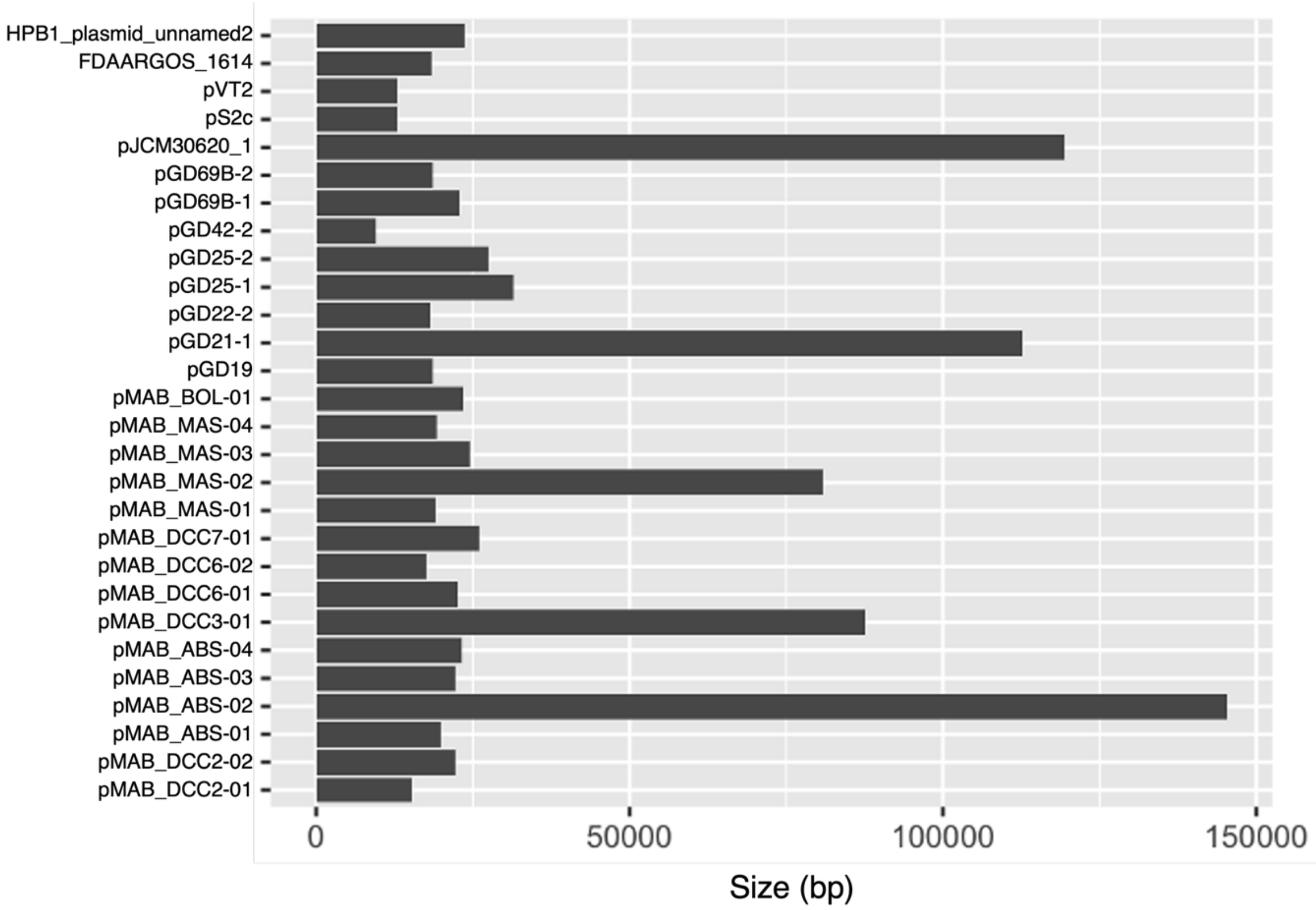

Supplementary figure 4. Size of identified plasmids in this study. Bar plot representing the size (in base pairs) of each identified plasmid across *M. abscessus* isolates. The y-axis lists the plasmid names, and the x-axis shows the size of each plasmid in base pairs.

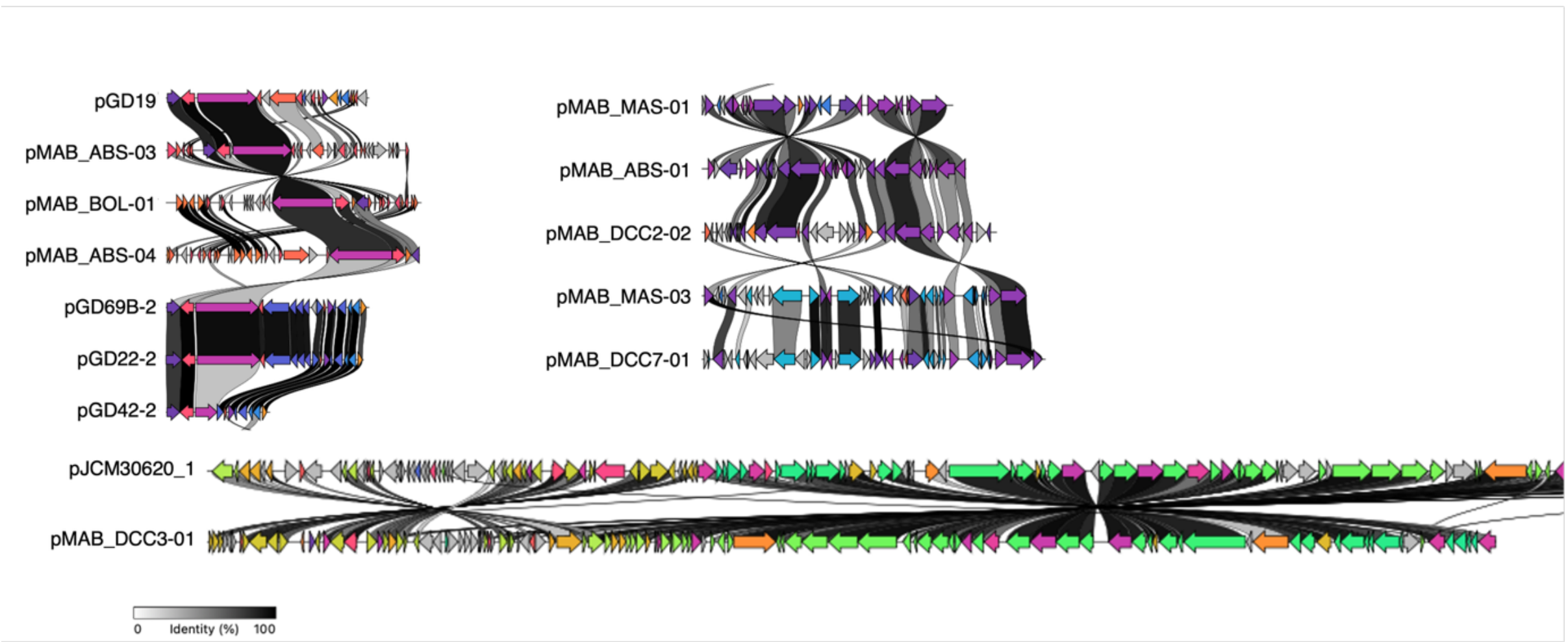

Supplementary figure 5. Gene synteny of plasmids. Arrows represent genes and their orientation, with colours denoting distinct genes. Connections between plasmids indicate homologous regions, with shading reflecting the degree of similarity.

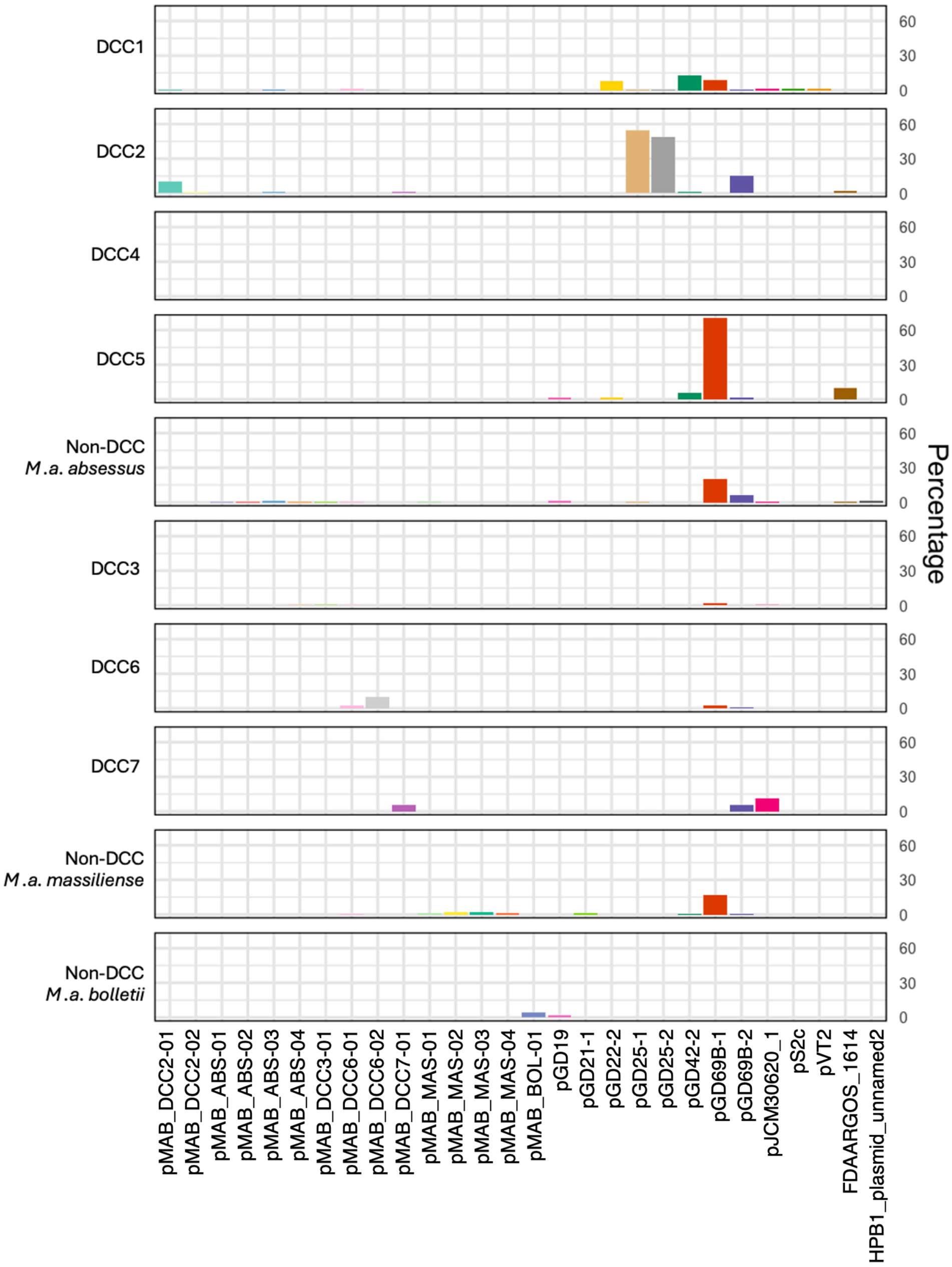

Supplementary figure 6. Proportion of isolates carrying plasmids in different *M. abscessus* DCCs. The plots show the percentage of isolates within each DCC that harbour plasmids. The x-axis lists the plasmid names, and the y-axis represents the percentage of isolates carrying each plasmid.

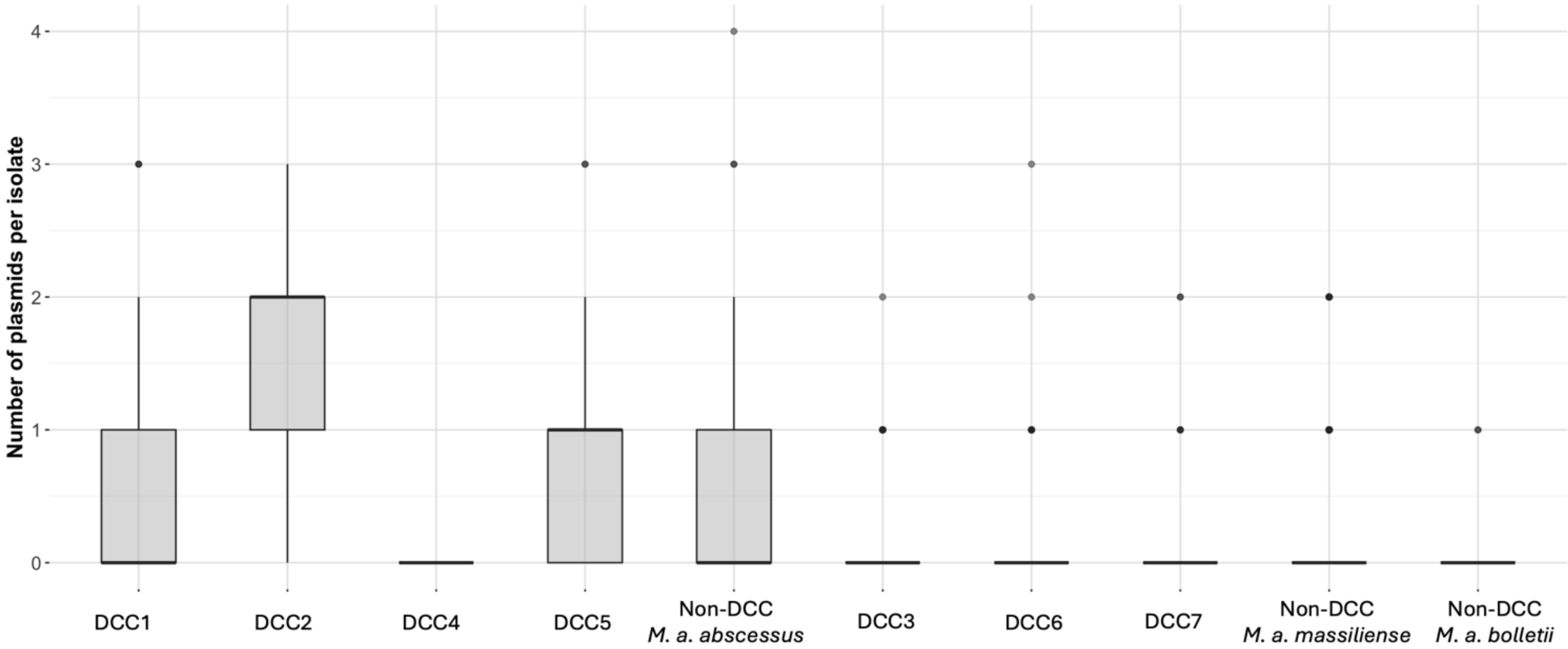

Supplement figure 7. Number of plasmids detected per isolate in different M. abscessus DCCs and non-DCC clades. The boxplots depict median and IQR with vertical lines extending from the boxes show the data range, up to 1.5 times the IQR, with data points beyond this range classified as outliers.

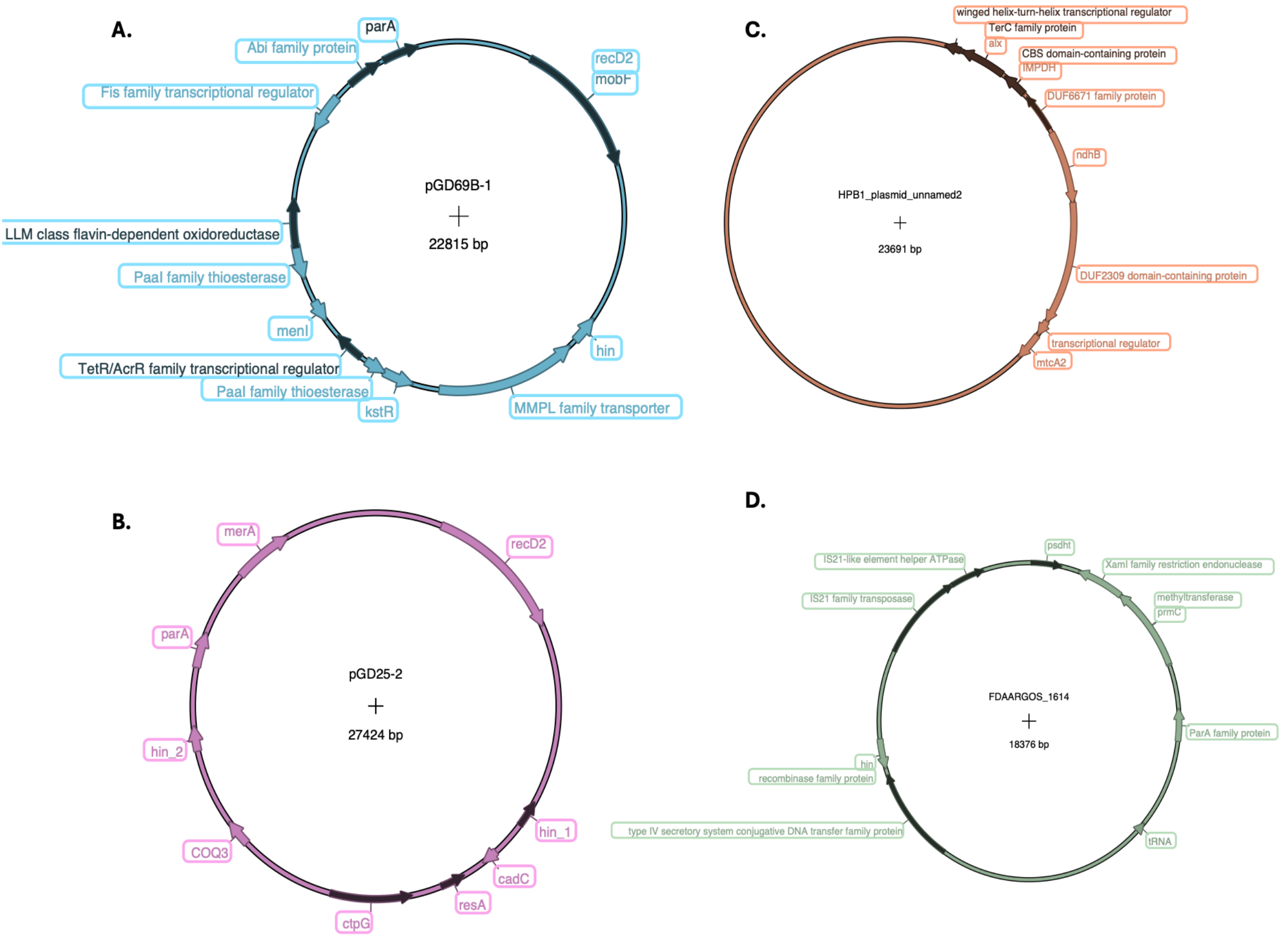

Supplement figure 8. Maps of plasmids with size and gene annotations: A) pGD69B-1; B) pGD25-2; C) HPB1_plasmid_unamed2 and D) FDAARGOS_1614. Gene annotation was performed using Prokka and integrated with annotations from the NCBI Prokaryotic Genome Annotation Pipeline. Plasmids were visualized using ApE.

Supplementary table 1. Plasmids detected by PlasmidFinder.

ID / Cluster	Sequence matched plasmid replicons (contig: start-end)	Plasmid	Previously found in
10665_2#81 / non-DCC *M. a. massiliense*	contig00125:1888-2364	pWBG738	*Staphylococcus epidermidis*
		pDLK1	*Staphylococcus epidermidis*
		pE5	*Staphylococcus aureus*
		pIM13	*Bacillus subtilis*
		pNE131	*Staphylococcus epidermidis*
20034_1#13 / non-DCC *M. a. massiliense*	contig00055:97-561	pSK6	*Staphylococcus aureus*
		pSK3	*Staphylococcus epidermidis*
12163_1#28 / non-DCC *M. a. abscessus*	contig00030:11193-12116	pKH17	*Staphylococcus aureus*
		SAP060B	*Staphylococcus aureus*
		pKC5b	*Lactobacillus fermentum*
		pUSA02	*Staphylococcus aureus*
		Cassette	*Staphylococcus aureus*
		pS0385-1	*Staphylococcus aureus*
		pWBG738	*Staphylococcus epidermidis*
		pDLK1	*Staphylococcus epidermidis*
		pE5	*Staphylococcus aureus*
		pIM13	*Bacillus subtilis*

Supplementary table 2. Coverage statistics and split-read support for representative plasmids identified in the study

Plasmid	Plasmid-associated genes	Representative id	Coverage - mean	Coverage - mode	#split paired reads within 200bp window of start or end
pMAB_ABS-01	RelB, RelE (toxin-antitoxin system)	ERR4020052	248.821	222	45
pMAB_ABS-02	RecD2, EccB5, EccC5 (ESX-5 secretion system)	ERR1081311	69.3698	68	3
pMAB_ABS-03	RecD2	12082_5#74	90.1709	85	15
pMAB_ABS-04	RecD2	SRR18969493	282.179	300	66
pMAB_BOL-01	RecD2	10665_2#62	85.1138	86	31
pMAB_DCC2-01	Soj (ParA-family partitioning ATPase), Hin, RecD2	10702_1#23	134.002	137	57
pMAB_DCC2-02	Hin	10665_2#80	107.744	105	31
pMAB_DCC3-01	RecD2, EccA2 (ESX-2 secretion system), SSB	DRR317480	167.805	168	7
pMAB_DCC6-01	Hin, RecD2	11893_6#70	137.258	132	25
pMAB_DCC6-02	ParA, Hin, RecD2	DRR245189	545.093	642	135
pMAB_DCC7-01	Hin	SRR7800425	203.057	96	1
pMAB_MAS-01	RecD2, EccA3 (ESX-3 secretion system)	SRR14719260	672.492	679	316
pMAB_MAS-02	Hin, RecD2	DRR245173	165.836	161	9
pMAB_MAS-03	RecD2	ERR2524316	248.134	247	34
pMAB_MAS-04	Hin, RecD2	DRR245164	160.373	158	11

Supplementary table 3. Summary of gene function annotation of identified plasmids.

Annotation	pMAB_DCC2-01	pMAB_DCC2-02	pMAB_ABS-01	pMAB_ABS-02	pMAB_ABS-03	pMAB_ABS-04	pMAB_DCC3-01	pMAB_DCC6-01	pMAB_DCC6-02	pMAB_DCC7-01	pMAB_MAS-01	pMAB_MAS-02	pMAB_MAS-03	pMAB_MAS-04	pMAB_BOL-01	pGD19	pGD21-1	pGD22-2	pGD25-1	pGD25-2	pGD42-2	pGD69B-1	pGD69B-2	pJCM30620_1	pS2c	pVT2	FDAARGOS_1614	HPB1_plasmid_unnamed2
Antitoxin	0	0	1	1	0	0	0	0	0	1	0	0	1	0	0	0	0	0	0	0	0	0	0	0	0	0	0	0
Toxin RelE	0	0	1	0	0	0	0	0	0	1	0	0	0	0	0	0	0	0	0	0	0	0	0	0	0	0	0	0
Single-stranded DNA-binding protein	0	0	0	0	0	0	1	0	0	0	0	1	0	0	0	0	0	0	0	0	0	0	0	1	0	0	0	0

Alcohol dehydrogenase	0	0	0	1	0	0	0	0	0	0	0	0	0	0	0	0	0	0	0	0	0	0	0	0	0	0	0	0
FAD-containing monooxygenase EthA	0	0	0	1	0	0	0	0	0	0	0	0	0	0	0	0	0	0	0	0	0	0	0	0	0	0	0	0
Glutathione reductase	1	0	0	0	0	0	0	0	0	0	0	0	0	0	0	0	0	0	0	0	0	0	0	0	0	0	0	0
Phosphonate dehydrogenase	0	0	0	0	0	0	0	1	0	0	0	0	0	0	0	0	0	0	0	0	0	0	0	0	0	0	0	0
NAD(P)H-quinone oxidoreductase subunit 2, chloroplastic	0	0	0	0	0	0	0	0	0	0	0	0	0	0	0	0	0	0	0	0	0	0	0	0	0	0	0	1
Mercuric reductase	0	0	0	0	0	0	0	0	0	0	0	0	0	0	0	0	0	0	0	1	0	0	0	0	0	0	0	0
Thiol-disulfide oxidoreductase ResA	0	0	0	0	0	0	0	0	0	0	0	0	0	0	0	0	0	0	0	1	0	0	0	0	0	0	0	0
Ubiquinone biosynthesis O-methyltransferase, mitochondrial	0	0	0	0	0	0	0	0	0	0	0	0	0	0	0	0	0	0	0	1	0	0	0	0	0	0	0	0
XamI family restriction endonuclease	0	0	0	0	0	0	0	0	0	0	0	0	0	0	0	0	0	0	0	0	0	0	0	0	0	0	1	0
DNA adenine methyltransferase YhdJ	0	0	0	0	0	0	0	0	0	0	0	0	0	0	0	0	1	0	0	0	0	0	0	0	0	0	0	0
Endoribonuclease MazF4	0	0	0	1	0	0	0	0	0	0	0	0	0	0	0	0	0	0	0	0	0	0	0	0	0	0	0	0
putative endoribonuclease MazF7	0	0	0	1	0	0	0	0	0	0	0	0	0	0	0	0	0	0	0	0	0	0	0	0	0	0	0	0
ESX secretion system	0	0	0	1	0	0	1	0	0	0	0	1	0	0	0	0	1	0	0	0	0	0	0	1	0	0	0	0
ESAT-6-like protein	0	0	0	1	0	0	0	0	0	0	0	0	0	0	0	0	0	0	0	0	0	0	0	0	0	0	0	0
Cytokinin riboside 5'-monophosphate phosphoribohydrolase	0	0	0	0	0	0	0	0	0	0	0	0	0	0	0	0	0	0	0	0	0	0	0	0	1	1	0	0
6-hydroxy-3-succinoylpyridine 3-monooxygenase HspA	1	0	0	0	0	0	0	0	0	0	0	0	0	0	0	1	0	0	0	0	0	0	0	0	0	0	0	0
6-hydroxy-3-succinoylpyridine 3-monooxygenase HspA	0	0	0	1	0	0	0	0	0	0	0	0	0	0	0	0	0	0	0	0	0	0	0	0	0	0	0	0
Hydroxymycolate synthase MmaA4	0	0	0	1	0	0	0	0	0	0	0	0	0	0	0	0	0	0	0	0	0	0	0	0	0	0	0	0
Long-chain-fatty-acid--CoA ligase	0	0	0	1	0	0	0	0	0	0	0	0	0	0	0	0	0	0	0	0	0	0	0	0	0	0	0	0
Inosine-5'-monophosphate dehydrogenase	0	0	0	0	0	0	0	0	0	0	0	0	0	0	0	0	0	0	0	0	0	0	0	0	0	0	0	1
1,4-dihydroxy-2-naphthoyl-CoA hydrolase	0	0	0	0	0	0	0	0	0	0	0	0	0	0	0	0	0	0	0	0	0	1	0	0	0	0	0	0

Thermonuclease	0	0	0	0	0	0	0	0	0	0	0	1	0	0	0	0	1	0	0	0	0	0	0	0	0	0	0	0
Mycosin-1	0	0	0	0	0	0	0	0	0	0	0	1	0	0	0	0	0	0	0	0	0	0	0	0	0	0	0	0
Mycosin-5	0	0	0	1	0	0	0	0	0	0	0	0	0	0	0	0	0	0	0	0	0	0	0	0	0	0	0	0
Non-haem bromoperoxidase BPO-A2	0	0	0	1	0	0	0	0	0	0	0	0	0	0	0	0	0	0	0	0	0	0	0	0	0	0	0	0
Phenylserine dehydratase	0	0	0	0	0	0	0	0	0	0	0	0	0	0	0	0	0	0	0	0	0	0	0	0	0	0	1	0
Putative membrane-bound redox modulator Alx	0	0	0	0	0	0	0	0	0	0	0	0	0	0	0	0	0	0	0	0	0	0	0	0	0	0	0	1
PaaI family thioesterase	0	0	0	0	0	0	0	0	0	0	0	0	0	0	0	0	0	0	0	0	0	1	0	0	0	0	0	0
Abi family protein	0	0	0	0	0	0	0	0	0	0	0	0	0	0	0	0	0	0	0	0	0	1	0	0	0	0	0	0
Glutaredoxin-like protein	0	0	0	1	0	0	1	0	0	0	0	0	0	0	0	0	0	0	0	0	0	0	0	0	0	0	0	0
L,D-transpeptidase 2	0	0	0	0	0	0	0	0	0	0	0	0	0	0	0	0	0	0	0	0	0	0	0	0	1	1	0	0
putative cysteine-rich protein YhjQ	0	0	0	0	0	0	0	0	0	0	0	0	0	0	0	0	0	0	0	0	0	0	0	0	1	1	0	0
Carbonic anhydrase 2	0	0	0	0	0	0	0	0	0	0	0	0	0	0	0	0	0	0	0	0	0	0	0	0	0	0	0	1
LLM class flavin-dependent oxidoreductase	0	0	0	0	0	0	0	0	0	0	0	0	0	0	0	0	0	0	0	0	0	1	0	0	0	0	0	0
Nucleoid-associated protein EspR	0	0	0	0	0	0	0	0	0	0	0	1	0	0	0	0	0	0	1	0	0	0	0	0	0	0	0	0
Aclacinomycin methylesterase RdmC	0	0	0	1	0	0	0	0	0	0	0	0	0	0	0	0	0	0	0	0	0	0	0	0	0	0	0	0
Mercuric resistance operon regulatory protein	1	0	0	0	0	0	0	0	0	0	0	0	0	0	0	0	0	0	0	0	0	0	0	0	0	0	0	0
ATP-dependent zinc metalloprotease FtsH	0	0	0	0	0	0	0	0	0	0	0	0	0	0	0	0	0	0	1	0	0	0	0	0	0	0	0	0
ImmA/IrrE family metallo-endopeptidase	0	0	0	0	0	0	0	0	0	0	0	0	0	0	0	0	0	0	1	0	0	0	0	0	0	0	0	0
Cadmium resistance transcriptional regulatory	0	0	0	0	0	0	0	0	0	0	0	0	0	0	0	0	0	0	0	1	0	0	0	0	0	0	0	0
Spermidine export protein MdtJ	0	0	0	1	0	0	0	0	0	0	0	0	0	0	0	0	0	0	0	0	0	0	0	0	0	0	0	0
putative cation-transporting ATPase G	0	0	0	0	0	0	0	0	0	0	0	0	0	0	0	0	0	0	0	1	0	0	0	0	0	0	0	0
MMPL family transporter	0	0	0	0	0	0	0	0	0	0	0	0	0	0	0	0	0	0	0	0	0	1	0	0	0	0	0	0
Phosphate-import ATP-binding protein PhnC	0	0	0	0	0	0	0	1	0	0	0	0	0	0	0	0	0	0	0	0	0	0	0	0	0	0	0	0
Phosphate-import	0	0	0	0	0	0	0	1	0	0	0	0	0	0	0	0	0	0	0	0	0	0	0	0	0	0	0	0

permease protein PhnE																												
putative phosphite transport system-binding protein PtxB	0	0	0	0	0	0	0	1	0	0	0	0	0	0	0	0	0	0	0	0	0	0	0	0	0	0	0	0
DNA-invertase hin	1	1	1	0	1	1	0	1	1	1	1	0	1	1	0	1	0	1	1	1	1	1	1	0	0	0	1	1
ATP-dependent RecD-like DNA helicase	1	0	0	1	1	1	1	1	1	0	0	1	0	1	1	1	1	1	0	1	1	1	1	1	1	1	0	0
Chromosome partitioning protein	1	0	0	0	0	0	0	0	1	0	0	0	0	1	0	1	0	0	1	1	0	1	0	0	0	0	1	0
Tyrosine recombinase XerD	0	0	0	1	0	0	0	0	0	0	0	0	0	0	0	0	0	0	0	0	0	0	0	0	0	0	0	0
IS21 family tranpoase	0	0	0	0	0	0	0	0	0	0	0	0	0	0	0	0	0	0	0	0	0	0	0	0	0	0	1	0
Transcriptional regulator WhiB	0	0	0	1	0	0	0	0	0	0	0	0	0	0	0	0	1	0	0	0	0	0	0	0	0	0	0	0
ECF RNA polymerase sigma factor SigR	0	0	0	1	0	0	0	0	0	0	0	0	0	0	0	0	0	0	0	0	0	0	0	0	0	0	0	0
HTH-type transcriptional regulator BetI	0	0	0	1	0	0	0	0	0	0	0	0	0	0	0	0	0	0	0	0	0	0	0	0	0	0	0	0
Putative transcriptional regulator of 2-aminoethylphosphonate degradation operons	0	0	0	0	0	0	0	1	0	0	0	0	0	0	0	0	0	0	0	0	0	0	0	0	0	0	0	0
RNA polymerase sigma factor RpoS	0	0	0	0	0	0	0	0	0	0	0	0	0	0	0	0	1	0	0	0	0	0	0	0	0	0	0	0
LuxR family transcriptional regulator	0	0	0	0	0	0	0	0	0	0	0	0	0	0	0	0	1	0	0	0	0	0	0	0	0	0	0	0
TetR/AcrR family transcriptional regulator	0	0	0	0	0	0	0	0	0	0	0	0	0	0	0	0	0	0	0	0	0	1	0	0	0	0	0	0
Fis family transcriptional regulator	0	0	0	0	0	0	0	0	0	0	0	0	0	0	0	0	0	0	0	0	0	1	0	0	0	0	0	0
Transcriptional repressor NrdR	0	0	0	0	0	0	0	0	0	0	0	0	0	1	0	0	0	0	0	0	0	0	0	0	0	0	0	0
HTH-type transcriptional repressor KstR	0	0	0	0	0	0	0	0	0	0	0	0	0	0	0	0	0	0	0	0	0	1	0	0	0	0	0	0
Alpha-D-ribose 1-methylphosphonate 5-triphosphate diphosphatase	0	0	0	0	0	0	0	1	0	0	0	0	0	0	0	0	0	0	0	0	0	0	0	0	0	0	0	0
Phthiocerol/phthiodiolone dimycocerosyl transferase	0	0	0	1	0	0	0	0	0	0	0	0	0	0	0	0	0	0	0	0	0	0	0	0	0	0	0	0

Putative oxidoreductase SadH	0	0	0	1	0	0	0	0	0	0	0	0	0	0	0	0	0	0	0	0	0	0	0	0	0	0	0	0
Release factor glutamine methyltransferase	0	0	0	0	0	0	0	0	0	0	0	0	0	0	0	0	0	0	0	0	0	0	0	0	0	0	1	0
Trans-aconitate 2-methyltransferase	0	0	0	0	0	0	0	0	0	0	0	0	0	0	0	0	0	0	1	0	0	0	0	0	0	0	0	0
type IV secretory system conjugative DNA transfer family protein	0	0	0	0	0	0	0	0	0	0	0	0	0	0	0	0	0	0	0	0	0	0	0	0	0	0	1	0

Supplementary table 4. Summary plasmid presence across longitudinal samples.

N = 304	Prevalence N (%)		Number of cases (%)	
**Plasmid**	**First timepoint**	**Longitudinal**	**Loss**	**Gain**
pGD69B-1	46 (15.1)	50 (16.4)	9 (18.4)	13 (26.0)
pGD25-1	25 (8.2)	23 (7.6)	4 (16.0)	2 (8.7)
pGD25-2	24 (7.9)	21 (6.9)	5 (20.8)	2 (9.5)
pGD69B-2	15 (4.9)	16 (5.3)	1 (6.7)	2 (12.5)
pGD42-2	14 (4.6)	15 (4.9)	0 (0.0)	1 (6.7)
pGD22-2	10 (3.3)	11 (3.6)	0 (0.0)	1 (9.1)
HPB1_plasmid_unnamed2	4 (1.3)	1 (0.3)	3 (75.0)	0 (0.0)
pMAB_DCC2-01	2 (1.3)	5 (2.3)	0 (0.0)	3 (42.9)
pGD19	3 (1.0)	2 (0.7)	1 (33.3)	0 (0.0)
pMAB_DCC2-02	1 (0.3)	0 (0.0)	1 (100.0)	0 (0.0)
FDAARGOS_1614	1 (0.3)	0 (0.0)	1 (100.0)	0 (0.0)
pMAB_DCC6-02	0 (0.0)	1 (0.3)	0 (0.0)	1 (100.0)
